# Lipoma of the finger presenting as restricted motion

**DOI:** 10.4103/0970-0358.44933

**Published:** 2008

**Authors:** Sukho Yoon, Sung-No Jung

**Affiliations:** Department of Plastic Surgery, The Catholic University of Korea, College of Medicine, Korea

Sir,

Although lipomas are common soft tissue neoplasms, they are rarely found in the fingers. Lipomas are often not considered in the initial differential diagnosis of digital swellings.[1]

**Figure 1:**
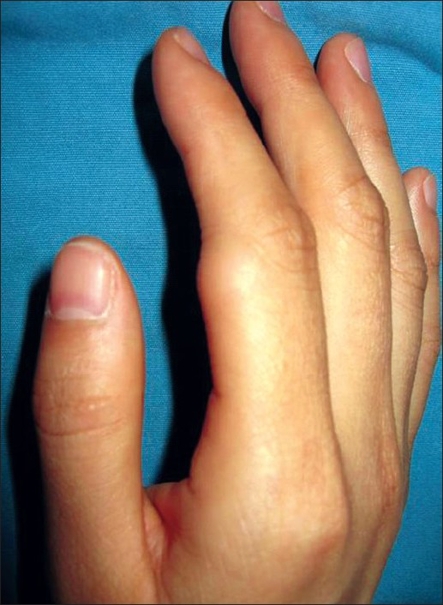
Our 35 year-old female patient with a nontender subcutaneous mass on the right index finger

**Figure 2:**
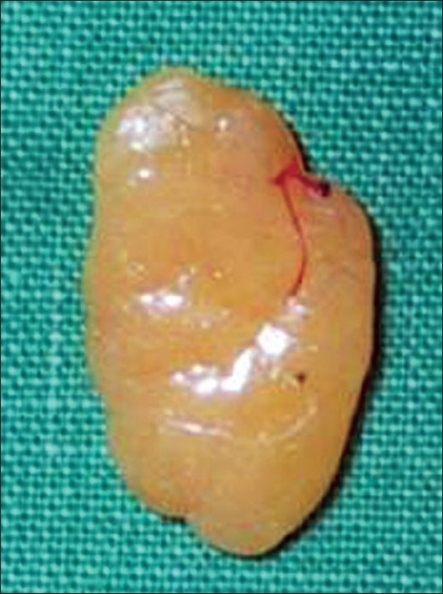
About 1.5 × 1cm sized lipomatous mass

**Figure 3:**
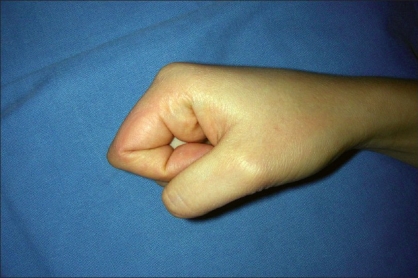
Thirty-six months after operation; there is no evidence of restriction

A 35 year-old female patient visited our outpatient clinic with a protruding skin lesion on the right index finger that caused discomfort with movement [Figure 1]. The patient reported that the lesion had started growing 12 months ago. Physical examination showed a 1.5 × 1.0 cm, nontender, subcutaneous mass on the radial side of the proximal phalanx associated with limitation of proximal interphalangeal joint motion; sensory symptoms were not present. The patient underwent an exploration of the finger under local anaesthesia using a longitudinal skin incision. There was no difficulty in excising the mass and its margins were free. The mass was excised completely and subjected to a histopathology examination. After surgery, the patient had fully recovered the motion of the finger. Grossly, the mass was 1.5 × 1.0 cm in size and was composed of fatty tissue [Figure 2]. Histological results showed that the mass was a lipoma. There was no evidence of recurrence or restriction in motion during the 36 months of postoperative follow-up and excellent range of motion [Figure 3].

In this rather unusual case, the lipoma was located on the proximal interphalangeal joint of the finger which restricted finger movement due to limited space.

Neoplastic lesions (liposarcoma, lipoblastoma, Giant cell tumour, spindle cell lipoma, angiolipoma, and neural fibrolipoma) and non neoplastic lesions (implantation cyst, pyogenic granuloma, and nodular fasciitis) with clinical characteristics similar to those of a lipoma of the finger should be considered in the differential diagnoses of a mass on the finger.[1] Ultrasonography, computerized tomography, or magnetic resonance imaging are useful for more detailed investigations and differential diagnosis.[2] Although a lipoma in the finger is relatively rare, it should be included in the initial differential diagnoses of finger tumours.
